# Can a community health worker and a trained traditional birth attendant work as a team to deliver child health interventions in rural Zambia?

**DOI:** 10.1186/s12913-014-0516-2

**Published:** 2014-10-27

**Authors:** Kojo Yeboah-Antwi, Davidson H Hamer, Katherine Semrau, Karen Z Waltensperger, Gail Snetro-Plewman, Chilobe Kambikambi, Amon Sakala, Stephen Filumba, Bias Sichamba, David R Marsh

**Affiliations:** Center for Global Health and Development, Boston University, 801 Massachusetts Ave, 3rd Floor, Boston, MA 02118 USA; Department of Global Health, Boston University School of Public Health, Boston, MA USA; Zambia Center for Applied Health Research and Development, Lusaka, Zambia; Save the Children, Department of Health and Nutrition, Africa Region, South Africa; Save the Children Zambia, Lusaka, Zambia; Save the Children, Westport, CT USA

**Keywords:** Teams, Teaming, Teamwork, Taskwork, Continuum of care, Community health workers, Traditional birth attendants, Newborn health, Child health care, Zambia

## Abstract

**Background:**

Teaming is an accepted approach in health care settings but rarely practiced at the community level in developing countries. Save the Children trained and deployed teams of volunteer community health workers (CHWs) and trained traditional birth attendants (TBAs) to provide essential newborn and curative care for children aged 0–59 months in rural Zambia. This paper assessed whether CHWs and trained TBAs can work as teams to deliver interventions and ensure a continuum of care for all children under-five, including newborns.

**Methods:**

We trained CHW-TBA teams in teaming concepts and assessed their level of teaming prospectively every six months for two years. The overall score was a function of both teamwork and taskwork. We also assessed personal, community and service factors likely to influence the level of teaming.

**Results:**

We created forty-seven teams of predominantly younger, male CHWs and older, female trained TBAs. After two years of deployment, twenty-one teams scored “high”, twelve scored “low,” and fourteen were inactive. Teamwork was high for mutual trust, team cohesion, comprehension of team goals and objectives, and communication, but not for decision making/planning. Taskwork was high for joint behavior change communication and outreach services with local health workers, but not for intra-team referral. Teams with members residing within one hour’s walking distance were more likely to score high.

**Conclusion:**

It is feasible for a CHW and a trained TBA to work as a team. This may be an approach to provide a continuum of care for children under-five including newborns.

## Background

Zambia has a strained health care system with limited health facilities and human resources, and thus has been using community-based health workers, mostly volunteers, to provide basic health services, especially in rural areas, to confront its high under-5 mortality [1,2]. Two common volunteer cadres are community health workers (CHWs) and trained traditional birth attendants (TBAs). CHWs as per government policy, have been trained to provide a wide range of services, including preventive and promotive interventions, health education, community mobilization and sensitization, and treatment of common childhood illnesses (fever, diarrhea, and pneumonia) [3]. Trained TBAs have also been trained to provide maternal and newborn interventions, including antenatal and postnatal care, and recognition of and referral for danger signs of pregnant women and newborns. As per government policy, TBAs are no longer trained in clean delivery but are encouraged to accompany women to health facilities to deliver. These community-based providers are supported by Neighborhood Health Committees (NHCs), which link them with both the community and the formal health system. The NHCs are community-based health management structures of community members with the responsibility of analyzing health situations and problems and exploring opportunities for solving them. Their roles include supporting community-based agents during implementation of health programs, initiating and supporting developmental activities to improve community and household health, and mobilizing community and local resources for health improvement. They represent communities on health center committees [3]. In this setting the NHCs play key roles in health delivery system and are seen as important partners by both the DHMT and the health center staff.

Trained TBAs and CHWs may reside in the same community, but work independently of each other, leading to inefficiency and missed opportunities for continuity of care. There is a growing recognition that health interventions for newborns should be integrated into child health programs [4] to promote a continuum of care, an approach expected to promote care for mothers and children from pregnancy to delivery, and into the immediate postnatal period and childhood [5].

*Teaming* (i.e., establishing teams of two or more individuals who work together) is an accepted approach in various settings, including health care, in both developed and developing countries; and it has increasingly become a critical approach in health care delivery [6-8], but it is not practiced with community-based health workers. Characteristically, team members generally have specialized knowledge and specific roles, make decisions, perform interdependent tasks, are adaptable, and share a common goal [9-11]. Benefits of a team include distributing workload, reinforcing individual capabilities, creating the feeling of participation and involvement, making better decisions, and generating a diversity of ideas for a common purpose [12]. *Teamwork* consists of behaviors related to team member interactions to achieve team goals, such as goal comprehension, communication, conflict management, decision-making/planning, leadership, mutual performance monitoring, mutual trust, team cohesion and team motivation [11,13-16]. Teamwork has increasingly been recognized by several organizations as important for improving healthcare [17-19]. *Taskwork*, on the other hand, consists of behaviors performed by individual team members to execute team functions [20,21].

Save the Children in collaboration with the Boston University Center for Global Health and Development (BU/CGHD) and the Ministry of Community Development, Mother and Child Health (MCDMCH), the Ministry of Health (MOH), and the Lufwanyama District Health Management Team (DHMT) is implementing the Lufwanyama Integrated Neonatal and Child Health Project in Zambia (LINCHPIN). LINCHPIN is an integrated, community-based newborn care and community case management package delivered through an enhanced district-wide community health program linked to health facilities and NHCs in a manner consistent with the Zambia MOH plans and policies and MCDMCH strategies and approaches [2,3]. The project teams CHWs and trained TBAs, supported by NHCs, to provide a continuum of evidence-based essential newborn and curative care for children 0–59 months of age in Lufwanyama District. The rationale for integration and teaming is to achieve efficiency, since the effect of the team will likely exceed the effects of the individuals working alone and also improve social cohesion and sense of community. This paper assessed whether CHWs and trained TBAs can work together in teams to provide integrated care to newborn and sick children in rural Zambia.

## Methods

### Study location

The study was conducted in Lufwanyama District in the Copperbelt Province of Zambia. Lufwanyama is a large, rural, undeveloped district with an estimated 2011 population of 87,592 [22], with the majority belonging to the Lamba ethnic group. Despite its location in the comparatively urban, industrialized Copperbelt, the district lacks physical infrastructure, and many roads are impassible during the rainy season. It has 12 health centers, five health posts and a newly opened district hospital. The DHMT operated for many years outside the district, but is currently housed at the new district hospital. Many basic health services, including treatment of minor illnesses, health education, antenatal care, family planning services, follow-up of patients with chronic illnesses and referrals, are provided through several categories of minimally trained community workers –trained TBAs, CHWs, male motivators, safe motherhood agents, family planning agents, disease surveillance agents, malaria agents, tuberculosis agents, HIV/AIDS agents, and untrained TBAs. The Lufwanyama DHMT, with support from non-governmental partners operating in the district, have trained and deployed CHWs and trained TBAs for over 30 years. CHWs and trained TBAs spend some days in a week working with health center staff at facilities. The health centers provide them with drugs and supplies and health workers supervise their work.

### Study design

This prospective study assessed the level of teamwork and taskwork among community-based CHW–TBA teams supported by NHC members. We used an assessment tool developed through formative research with community leaders, health workers, CHWs and trained TBAs [23]. We carried out the assessment every six months from June 2011 to March 2013.

### Team creation and training

A CHW and trained TBA working in the same community formed the CHW-TBA team. We did not create teams for communities which had only a CHW or trained TBA. The CHW-TBA team plus two NHC support members were trained in teaming concepts prior to deployment. The training addressed both specific tasks (Table 1) that the teams would undertake as well as the skills and competencies to maintain a functioning team**.** The teamwork skills and competencies included i) good communication; ii) respectful dialogue and action; iii) each helping the other, mutual support, and working hand in hand; iv) assess, make decisions and manage conflicts; v) trust and confidentiality of care-seekers/community members; vi) together monitor team task and team maintenance abilities; vii) evaluate successes and failures; viii) asking for feedback; and ix) motivate and encourage each other. The specific tasks and skills required for successful community teams were identified during earlier formative research [23].The training emphasized the importance of performing the joint tasks and the need to document tasks performed. They practiced and demonstrated how to perform these tasks. The training utilized several methods including exercises, practice, demonstrations, role play, experience sharing brainstorming and real-life scenarios for the teams to acquire the necessary knowledge and skills of teamwork competences for maintaining functioning teams. The training also clarified roles of the NHCs as identified by the MOH guidelines [3].

**Table 1:** **Taskwork description**

**Task**	**Description**
Meeting with NHCs	This task requires the team to meet with NHC to discuss CHW/TBA team work and performance including challenges and the support needed.
Conducting behavior change communication (BCC)	Sessions in the community to educate community members in relevant health topics including exclusive breastfeeding, disease prevention, danger signs in pregnancy and childhood illnesses, importance of antenatal and postnatal care, hygiene and sanitation.
Problem solving for newborn and child care	Home visits including follow-up visits to help and support caregivers in their care of children such as individual counselling, addressing challenges and seeking care
Outreach services	Publicizing dates of outreach, mobilizing caregivers to attend and performing specific activities during sessions.
Support Referral	Convincing caregivers and households on the need to accept a referral and help with mobilizing transport
Intra-team referral	CHW referring pregnant or postnatal women seen at clinic or during a home visit to the trained TBA for follow-up. Trained TBA referring sick child seen on home visit or at postnatal care to CHW for treatment and advice.
Postnatal care visit at 6–8 weeks	Joint home visits to children aged 6–8 weeks in order for the trained TBA to “hand over” care of child to the CHW.

### Baseline data collection

Prior to training, we collected baseline information from team members, including age, gender, education, ethnic group, marital status, religion, membership of a social group (e.g. faith-based fellowships, parent-teacher associations, corporative societies, etc.), length of service, other occupation, and walking time from each other.

### Team assessment

An independent, non-LINCHPIN data collector visited the core team members (CHW and trained TBA) and administered a three-part team measurement tool. The first assessment started two months after the teaming training. Part A was administered to both members together and assessed taskwork, i.e., whether the team had jointly performed any of seven agreed specific tasks in the previous three months: 1) meeting with NHCs to discuss work and performance, 2) conducting behavior change communications sessions targeting women on newborn and child care, 3) problem-solving for newborn or child care, 4) participating in outreach services, 5) supporting referral of a pregnant woman or sick child, 6) conducting intra-team referral, and 7) conducting postnatal care visits to a mother with a newborn aged 6–8 weeks. The team scored “0” if a function was not performed, “1” if performed but without documented evidence, and “2” if there was documented evidence of performance. Part B was administered separately to the CHW and trained TBA. It assessed 27 characteristics from eight teamwork processes identified during the formative research [23]: 1) mutual performance monitoring, 2) mutual trust, 3) decision making/planning, 4) team cohesion, 5) team motivation, 6) goals and objectives, 7) communication, and 8) conflict resolution/management. Data were collected from each member about whether, in his/her opinion, the characteristic was present in the team over the previous six months. They scored “1” if a member reported that the characteristic was not or hardly present in the team; “2” if it was present sometimes; and “3” if present all the time. The score for the team was the average score of the two members. Part C – also administered separately to each individual team member – collected information on perceived factors that may influence teamwork such as supervision, refresher training, availability of supplies, incentives, and ownership of bicycle or cell phone.

### Team score and classification and analysis

The score for the taskwork of each team at each assessment was the sum of the scores of the seven functions. The overall taskwork score for the teams was the mean score of the four assessments. For teamwork, the score for the team at each assessment was the average score of the two members from the twenty seven indicators (expressed as a percent). The overall teamwork score was also the mean score of the four assessments.

A team was categorized “inactive” if unavailable for an assessment and the local NHC confirmed its inactivity and break-up. We categorized the remaining teams “high” if the mean score on the taskwork scale was ≥7 of a possible 14, and the mean score on the teamwork scale was ≥90%; and “low” if the taskwork score was <7 or teamwork score was <90%. We decided on the cut-offs prior to data collection, but we modified the categorization based on the distribution of the teamwork scores. In order to evaluate factors that may influence the level of teaming, frequency and proportions were compared with chi-square test; odds ratios (OR) and 95% confidence intervals (CI) were calculated for each characteristic. All data analysis was conducted in EpiInfo software package [24].

### Ethical issues

Ethical approval was obtained from the Boston University Institutional Review Board (BU-IRB) and a local Zambian ethical review committee (ERES CONVERGE). Informed consent was obtained from all study participants with a consent form developed in accordance with guidelines of the BU-IRB and the local ethical review committee and translated into Bemba, the language of common communication in the district. During consenting, study personnel explained the purpose and rationale of the study, informed the participants that they were not obliged to participant in the research, and assured them of the confidentiality of the information collected from them.

## Results

### Team characteristics

The project created and trained 47. There were 74 CHWs operating in the district but some CHWs did not have a trained TBA operating in their communities. The CHWs were predominantly male (80.9%), and the trained TBAs were all female (Table 2). CHWs were younger than the trained TBAs (average age of 44 years vs 53 years). Most CHWs had more schooling than the trained TBAs. Half the trained TBAs were of the local Lamba ethnic group while only a third of the CHWs were Lamba. CHWs were more likely to be currently married than the trained TBAs. Only about a fifth of the CHWs and the trained TBAs reported that being a CHW or TBA was their main occupation.

**Table 2:** **Baseline characteristics of team members**

**Characteristics**	**CHW (n = 47)**	**TBA (n = 47)**
***Age: (years)***		
Average (SD)	44.4 (8.8)	53.0 (6.6)
Range	28 – 69	33 – 66
***Sex:***		
Male	80.9%	0
Female	19.1%	100%
***Educational Level***		
No education	0	8.5%
Primary	14.9%	68.1%
Secondary	85.1%	23.4%
***Ethnic Group***		
Lamba	36.2%	50%
Bemba	14.9%	16.5%
Kaonde	2.1%	2.2%
Other	46.8%	41.3%
***Marital status***		
Single/not married	0	2.1%
Married	91.3%	66.0%
Separated/divorced	2.2%	6.4%
Widowed	6.5%	25.5%
***Religion***		
Christian (Jehovah witness)	31.9%	19.2%
Christian (Catholic)	12.8%	10.6%
Christian (Pentecostal)	6.4%	10.6%
African Christian Church	25.5%	44.7%
Other	23.4%	14.9%
***Main Occupation***		
CHW/TBA	23.9%	19.2%
Farmer	76.1%	80.8%
***Length of Service (years)***		
Average (SD)	9 (5.9)	11.3 (7.7)
Range	1-28	3-40

### Overall team categorization

We categorized 21 (44.7%) teams as high, 12 (25.5%) as low, and 14 (29.8%) as inactive. Three teams became inactive after the first assessment, four after the second, and the remaining seven after the third. CHW departure, usually to find a new job, was responsible for most of the inactive teams (71.4%) (Table 3). Two CHWs were employed as casual laborers to work at rural health centers, two CHWs stopped because they became frustrated with the work and one trained TBA was forced to stop because some members of the community believed she was not representing community values.

**Table 3:** **Reasons for inactive teams**

**Reason**	**CHW (n = 47) n (%)**	**TBA (n = 47) n (%)**	**Total (n = 94) n (%)**
Found new job	5 (10.6)	0	5 (5.3)
Relocated to another area	2 (4.3)	2 (4.3)	4 (4.3)
Illness/old age	1 (2.1)	1 (2.1)	2 (2.1)
Frustration	2 (4.3)	0	2 (2.1)
Forced to stop	0	1 (2.1)	1 (1.1)
Total	10 (21.3)	4 (8.5)	14 (14.9)

### Teamwork performance

**Figure 1:**
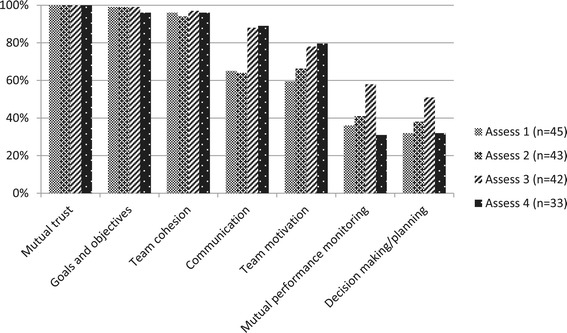
**Teamwork performance – proportion of teams that exhibited teamwork processes during assessments.**

All team members reported the presence of mutual trust within their teams during all four assessments (Table 4). Many team members reported comprehension of team goals and objectives and team cohesion as present most of the time. On the other hand, decision making/planning and mutual performance monitoring were reported lacking in most cases. The teams reported only six conflicts in the four assessments, all of which were satisfactorily resolved or managed. Team motivation and communication were reported to have improved over time while mutual performance monitoring and decision making/planning after initial improvement, declined during the last assessment (Figure 1).

**Table 4:** **Teamwork Performance – proportion of teams that exhibited teamwork processes during the four assessments**

**Teamwork dimension**	**Average performance**
Mutual trust	100%
Goals and objectives	98.1%
Team cohesion	95.7%
Communication	76.3%
Team motivation	70.8%
Mutual performance monitoring	41.3%
Decision making/planning	38.1%

### Taskwork performance

**Figure 2:**
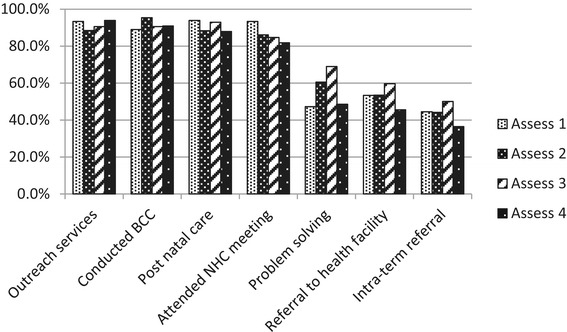
**Taskwork performance – proportion of teams that performed the identified tasks during assessments.**

Table 5 shows reported and documented joint activities. The most common *documented* joint activity was making a home visit to a mother with a young infant aged about 6–8 weeks where the trained TBA “handed over” the child to the CHW (55.3%), followed by meeting with NHCs to discuss work and performance (36.5%). Less commonly reported joint activities were intra-team referral (e.g., the CHW referring a pregnant woman to the trained TBA or the trained TBA referring a mother with a sick child to the CHW) and joint problem solving (15.6 and 21.6%, respectively). The most common joint activity (documented plus undocumented) was participation in outreach services, including immunization conducted by the supervising rural health center staff, and BCC sessions targeting women to educate them about newborn and child care (Figure 2). The least common activities by these criteria were intra-team referral and supporting referral to health facilities.

**Table 5:** **Taskwork – proportion of teams that performed the agreed task during the four assessments**

**Taskwork**	**Average performance (documented)**	**Average performance (undocumented)**
Attended NHC meeting	36.5%	50.3%
Conducted BCC	31.2%	60.3%
Problem solving	21.6%	34.5%
Outreach services	21.8%	69.8%
Referral to health facility	28.1%	24.9%
Intra-team referral	15.5%	28.3%
Post natal care	55.3%	35.3%

### Factors influencing teaming

Teams with members residing within one hour’s walking distance were more likely to score high (OR = 5.80; 95% CI: 1.52-22.1; p = 0.007). Teams whose members were jointly supervised were also more likely to score high (OR = 3.2; 95% CI: 0.83-12.74; p = 0.05), barely achieving statistical significance (Table 6). Teams whose members were of the same sex and with at least one member receiving some form of incentives (e.g. payment in-kind or cash from the community for services rendered) were likely to score high, but these differences were not significant.

**Table 6:** **Determinants of level of teaming**

**Determinants**	**Team Status**		
	**High (N = 21) n (%)**	**Low/Lost (N = 26) n (%)**	**High vs Low/Lost OR (95% CI), p-value**
Same gender	5 (23.8)	4 (15.3)	OR = 1.72 (0.40-7.43) p =0.36
Same educational level	10 (47.6)	14 (53.8)	OR = 0.78 (0.25-2.47) p =0.44
Same tribe	6 (28.6)	6 (23.1)	OR = 1.33 (0.36-5.00) p =0.46
Same marital status	14 (66.7)	13 (50.0)	OR = 2.0 (0.61-6.57) p =0.20
Same religion	8 (38.1)	10 (38.5)	OR = 0.98 (0.30-3.21) p =0.61
Belonging to a social group	9 (42.9)	11 (42.3)	OR = 1.02 (0.32-3.27) p =0.60
Supervised together some of the time^1^	14 (66.7)	10 (38.5)	OR = 3.2 (0.96-10.66) p = 0.05
Team member received some form of payment^2^	10 (47.6)	7 (26.9)	OR = 2.46 (0.73-8.34) p =0.12
Team member owes bicycle most of the time^3^	21 (100)	24 (92.3)	OR = Undefined p = 0.30
Team members owe mobile phone most of time^4^	3 (14.3)	5 (19.2)	OR = 0.70 (0.15-3.34) p =0.48
Team members have combined meeting with community most of time^5^	18 (85.7)	22 (84.6)	OR = 1.09 (0.21-5.52) p =0.62
Team members have supplies most of time^6^	1 (4.8)	3 (11.5)	OR = 0.38 (0.04-3.98) p =0.39
Team members some primary occupation apart from being CHW/trained TBA	9 (42.9)	7 (26.9)	OR = 2.04 (0.60-6.9) p =0.20
Team members within an hour walking distance	17 (80.9)	11 (42.3)	OR = 5.80 (1.52-22.1) p =0.007

^1^ Both the CHW and trained TBA reported being supervised together at least once during the assessments.

^2^ At least one member (either the CHW or trained TBA) reported receiving some form of payment (cash, kind or both) at least once during the assessments

^3^ At least one member of the team (CHW or trained TBA) reported owing a bicycle more than half of the assessments.

^4^ Both the CHW and trained TBA reported owing mobile phones more than half of the assessments.

^5^ Both the CHW and trained TBA reported having meeting together with the community leaders more than half of the assessments.

^6^ Both the CHW and trained TBA reported having the needed supplies to work with more than half of the assessments.

## Discussion

This study shows the feasibility of creating and deploying teams of volunteer community-based providers of relatively younger, better schooled, predominantly male CHWs and older, less schooled, female trained TBAs in a rural setting. Most of the important teamwork dimensions – i.e., mutual support, team cohesion, comprehension of team goals and objectives and communication [6,11,25] – were highly present in the teams. Additionally, most teams performed many of the joint tasks. About two-thirds of the active teams were categorized as high performing.

Having a common purpose that all team members are able to articulate is fundamental to team effectiveness. Teams need to involve all members in purpose development, and everyone should be able to articulate and commit to the team’s purpose. If team members have different understandings of what their common purpose is, friction, confusion, and wasted resources and effort are inevitable [26]. In our study, team scores on the comprehension of goal and objectives were high; therefore, these CHW-TBA teams had the potential to be effective in delivering integrated newborn and child care services in a rural setting. Team scores on communication were also high and improved over time, a welcome achievement since team communication failure has been associated with breakdown of teamwork, reduced outcomes, tension, stress and inefficiency [27-32].

The low score for mutual performance monitoring is of great concern. A proposed model of five key dimensions for effective teams includes mutual performance monitoring [33]. Mutual performance monitoring requires sufficient understanding of the environment to monitor other team members to identify lapses. To achieve these five dimensions, team members must respect and trust each other to give and receive performance feedback and must have good communication skills to convey information accurately [34]. Despite scoring low in mutual performance monitoring, these teams had excellent scores on mutual trust and high scores on communication, so these teams have the potential to improve monitoring.

Postnatal care coverage is low in Zambia, and newborns in rural areas are less likely to have postnatal care especially within the critical first week of life than newborns in urban areas [2]. It was reassuring that one of the most commonly performed tasks was the trained TBA and CHW jointly making home visits for handover at 6–8 weeks. CHWs normally see infants from two months of age and trained TBAs are supposed to carry out home visits soon after a baby’s delivery, encourage facility-based postnatal care, assess for danger signs in mother and baby, and make and follow up referrals when necessary. The joint home visits for handing over care of the young infant has the potential to underscore the importance of and improve the use of facility-based postnatal services and enhance the continuum of care. It is possible that the high performance of this task was because it was related to the responsibilities of both team members (to make home visits) and therefore it was easier to undertake joint activities that are already perceived to be part of their routine activity.

CHW-TBA teams appear to be a viable strategy to implement an integrated community-based newborn and child care interventions; however, 30% team attrition over two years presents a challenge. This is not surprising considering that many teams received few or no incentives from their communities. Annual attrition rates as high as 77% have been reported among volunteer community-based providers [35]. Attrition is largely due to low remuneration, “movement upwards to higher positions in the health system,” and finding better positions in other fields [36], similar to what we found. The importance of adequate retention and incentive structures for CHW programs is recognized as a key component of the WHO task-shifting proposal to tackle health worker shortages to contribute to the achievement of several Millennium Development Goals in low-income countries [37]. If teaming is to be implemented, approaches to motivate and retain CHWs need to be adopted [38-41]. The development and implementation of the Zambian government’s new National Community Health Worker Strategy, which established a new cadre of community health assistants who will be paid a monthly allowance by the government, may be a step in the right direction [2].

Member proximity was the main identified factor positively influencing the level of teaming. It is not surprising since this situation is likely to improve the communication and interaction between team members and thereby improve collaborative efforts.

### Limitations

The study has limitations. The assessment consisted mainly of participants’ subjective reports of satisfaction, attitudes, and opinions, and they may have over-rated themselves. The small sample size may have precluded identifying other factors influencing teaming. Another limitation was that the assessment tool was not validated.

## Conclusions

To our knowledge, this is the first attempt to assess the feasibility of community-based teams in a health care setting in a developing country. We measured teamwork using culturally accepted relevant teamwork dimensions and agreed upon tasks the teams were expected to perform. The teams’ performances on both the teamwork and taskwork scales were encouraging. Creating, supporting, measuring and adapting teams have the potential to strengthen community capacity to improve health delivery. Communities provide the social, cultural and organizational support and allocate and manage resources to address challenges that affect their members. Teaming is likely a promising potentially sustainable approach to deliver continuous newborn and child health interventions in rural communities and may accomplish development in other sectors. The DHMT, health center staff, community leaders and members, CHWs and trained TBAs were actively involved in the development of the tool. LINCHPIN has started discussion with the DHMT about incorporating the teaming approach in the health delivery system.

## Abbreviations

BU-IRB: Boston University Institutional Review Board
CGHD: Center for Global Health and Development
CHW: Community health worker
CI: Confidence interval
DHMT: District Health Management Team
LINCHPIN: Lufwanyama Integrated Neonatal and Child Health Project
MCDMCH: Ministry of Community Development, Mother and Child Health
MOH: Ministry of Health
NHC: Neighborhood Health Committee
OR: Odds ratio
TBA: Traditional birth attendant
